# A Smart Mobile App to Simplify Medical Documents and Improve Health Literacy: System Design and Feasibility Validation

**DOI:** 10.2196/35069

**Published:** 2022-04-01

**Authors:** Rasha Hendawi, Shadi Alian, Juan Li

**Affiliations:** 1 North Dakota State University Fargo, ND United States

**Keywords:** health literacy, knowledge graph, natural language processing, machine learning, medical entity recognition

## Abstract

**Background:**

People with low health literacy experience more challenges in understanding instructions given by their health providers, following prescriptions, and understanding their health care system sufficiently to obtain the maximum benefits. People with insufficient health literacy have high risk of making medical mistakes, more chances of experiencing adverse drug effects, and inferior control of chronic diseases.

**Objective:**

This study aims to design, develop, and evaluate a mobile health app, MediReader, to help individuals better understand complex medical materials and improve their health literacy.

**Methods:**

MediReader is designed and implemented through several steps, which are as follows: measure and understand an individual’s health literacy level; identify medical terminologies that the individual may not understand based on their health literacy; annotate and interpret the identified medical terminologies tailored to the individual’s reading skill levels, with meanings defined in the appropriate external knowledge sources; evaluate MediReader using task-based user study and satisfaction surveys.

**Results:**

On the basis of the comparison with a control group, user study results demonstrate that MediReader can improve users’ understanding of medical documents. This improvement is particularly significant for users with low health literacy levels. The satisfaction survey showed that users are satisfied with the tool in general.

**Conclusions:**

MediReader provides an easy-to-use interface for users to read and understand medical documents. It can effectively identify medical terms that a user may not understand, and then, annotate and interpret them with appropriate meanings using languages that the user can understand. Experimental results demonstrate the feasibility of using this tool to improve an individual’s understanding of medical materials.

## Introduction

### Background

Effective communication in health care has an enormous impact on the health and safety of patients. Limited health literacy is one of the major obstacles to good health care results including health status, health outcomes, health care use, and health costs for patients [1]. Health literacy is “the degree to which individuals have the capacity to obtain, process, and understand basic health information and services needed to make appropriate health decisions” [2]. In today’s health care systems, patients are expected to read long lists of complex health care documents, such as detailed home care guidelines, medication information, consent forms, discharge instructions, insurance summaries, and health educational materials. Misunderstanding of such information can lead to negative results. Unfortunately, many of these materials are difficult to understand. New medical achievements have introduced new jargon, descriptions, and medical terminologies, making it even more difficult to comprehend, even for individuals with sufficient literacy. Studies have shown that people with insufficient health literacy know less about their illness, lack proper health self-management knowledge, and have few precautionary measures for their health [3].

However, according to the US Department of Health and Human Services, only 12% of adults in the United States have proficient health literacy, whereas more than one-third of adults have low health literacy levels, which make it difficult for them to deal with common health tasks such as following directions for how to use prescription medications [4]. Low health literacy is a serious problem, especially in underrepresented racial or ethnic groups and older adults [4]. For example, the proportion of adults with basic or below basic health literacy ranges from 28% among White adults to 65% among Hispanic adults [5]. Adults aged ≥65 years are more likely to have below basic or basic health literacy skills than those aged <65 years. The proportion of adults at these lower levels of literacy was greatest for those aged >75 years [4]. Centers for Disease Control has been engaged in the plain language effort to encourage communication effectively in culturally appropriate ways. Although using plain language is a promising idea, many organizations do not use it as often as they should [6].

### Objectives

Given the aforementioned gap between the current health information and people’s poor understanding of this information to make life-altering decisions, many policies and strategies have been proposed by policy makers, administrators, educators, and health care professionals to simplify medical information and improve health literacy. Besides these efforts, there is an increasing need to provide tools to facilitate people to understand medical information. This may enhance the patient-physician relationship and improve health care outcomes by reducing the incidence of morbidity, mortality, and misuse of health care [7]. For this purpose, in this paper, we propose a mobile health (mHealth) app to help users understand complex medical documents and improve their health literacy. On the basis of a user’s health literacy level, the tool will translate into or interpret a complex medical document in languages that the user is familiar with and at appropriate reading levels. Evaluation surveys are provided to users to evaluate the effectiveness of this tool and the users’ satisfaction. This tool will help to make health information accurate, accessible, and actionable.

## Methods

### Ethics Approval

This study was approved by the institutional review board of North Dakota State University (IRB0003857).

### System Overview

**Figure 1:**
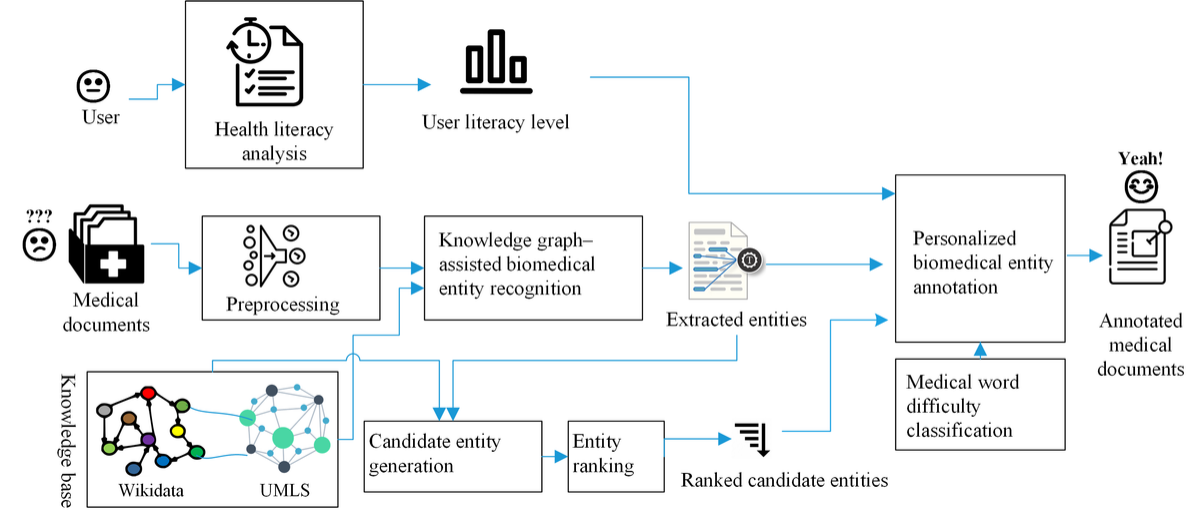
The architecture of the system. UMLS: Unified Medical Language System.

The goal of the system is to design a mobile app to remove people’s barriers to understanding difficult medical documents by annotating or interpreting medical terminologies with plain texts, which they can understand easily. The app, MediReader, is built based on comprehensive knowledge sources and artificial intelligence–based processing mechanisms. It annotates a medical document with external knowledge according to each user’s health literacy level. Figure 1 illustrates the architecture of the proposed system. First, MediReader identifies a user’s health literacy level. Then, it annotates the documents such that it is tailored to the user’s skill level. Medical terms will be identified with the help of external medical dictionaries. Then, based on the user’s health literacy level, *complex* medical entities will be linked to and explained by entities in the external knowledge base or data set. The complexity of a term is relative to the specific user; therefore, users with different health literacy levels may obtain different annotation results. We present the details of the system components in the following subsections.

### Knowledge Base Construction

We created a comprehensive medical knowledge base by integrating multiple publicly available knowledge sources, including Unified Medical Language System (UMLS) [8] and Wikidata [9]. Specifically, we used UMLS’s three knowledge resources: Metathesaurus, semantic network, and specialist lexicon and lexical tools. Vocabularies gathered in the UMLS Metathesaurus include the National Center for Biotechnology Information taxonomy [10], gene ontology [11], Medical Subject Headings [12], Online Mendelian Inheritance in Man [13], Digital Anatomist Symbolic Knowledge Base [14], Systematized Nomenclature of Medicine–Clinical Terms [15], International Classification of Diseases and Health-Related Problems–10th edition [16], Medical Dictionary for Regulatory Activities [17], and others. Wikidata is a multidisciplinary ontological database that encompasses many medicine-related entries such as human genes, human proteins, diseases, drugs, drug classes, therapies, human arteries, human muscles, human nerves, medical specialties, surgical procedures, human veins, pains, human bones, human enzymes, syndromes, human joints, and human ligaments.

### User Health Literacy Measurement

The objective of our health literacy measurement was to identify the degree to which individuals can understand health information and services. We studied many health literacy screening and measurement approaches, including the National Assessment of Adult Literacy [4], Rapid Estimate of Adult Literacy in Medicine [18], Test of Functional Health Literacy in Adults [19], Newest Vital Sign [20], Wide Range Achievement Test [21], ComprehENotes [22], and so on. We adopted the recently proposed approach, ComprehENotes, as our literacy screening approach, as its questions are sufficiently general to be applicable to a wide variety of individuals while still being grounded in specific medical concepts. Most of the questions have low difficulty estimates, which makes the test appropriate for screening for low health literacy. We chose questions from the question set of ComprehENotes that is created from real patients’ electronic health records (EHRs) [22]. Experts including physicians and medical researchers identified important concepts from the EHR of six common diseases (heart failure, diabetes, cancer, hypertension, chronic obstructive pulmonary disease, and liver failure). Medical experts believe that these concepts are important for patients to understand the EHR materials. The test questions were designed to assess the comprehension of these concepts.

We chose a subset of ComprehENotes’ questions to perform user evaluation, as a test with fewer questions can be administered more quickly than the full test. The subset of the questions should be sufficiently informative to identify different health literacy levels. We used the item response theory (IRT) [23] to choose a good subset of questions. IRT models the relationship between latent traits (unobservable characteristics or attributes) and their manifestations (ie, observed outcomes, responses, or performance) [24]. IRT has been widely used to analyze individuals’ responses (graded as right or wrong) to a set of questions. IRT predicts the performance of a test by jointly modeling individual ability and item characteristics. Using IRT, we repeatedly removed questions that cannot distinguish between individuals with high ability levels and individuals with low ability levels. Then, we identified n (n<55) questions from the original 55 questions with the largest discrimination capability and highest average information for inclusion in the short form of the test to make it as informative as possible.

ComprehENotes uses the IRT model that is widely used in education to calibrate and evaluate items in tests, questionnaires, and other instruments and to score participants on their abilities, attitudes, or other latent traits. Specifically, we applied the 3-parameter logistic model, in which the item characteristic curves are assumed to follow a logistic function with a nonzero lower asymptote:

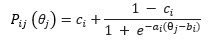



In the above equation, Pij is the probability that person j answers item i correctly, and θj is the ability level of individual j. In our project, θ represents the ability of an individual in the task of medical document comprehension. As individuals are assumed to be sampled from a population, their ability levels are assumed to have a random effect with a standard normal distribution. Therefore, a score of 0 is considered as average (ie, in the 50th percentile), scores >0 are considered as above average, and scores <0 are considered as below average.

### Medical Entity Identification

In this task, medical entities in a document, such as diseases, medical problems, drug names, tests, and examinations, will be identified. Existing research on biomedical named entity recognition can be classified into three types: rule-based [25], dictionary-based [26], and machine learning–based approaches [27]. Machine learning–based approaches are more accurate and stable than rule-based and dictionary-based approaches, as machine learning–based approaches have the potential to manage features with high dimensions and find new terms and variants based on the learning trends.

**Figure 2:**
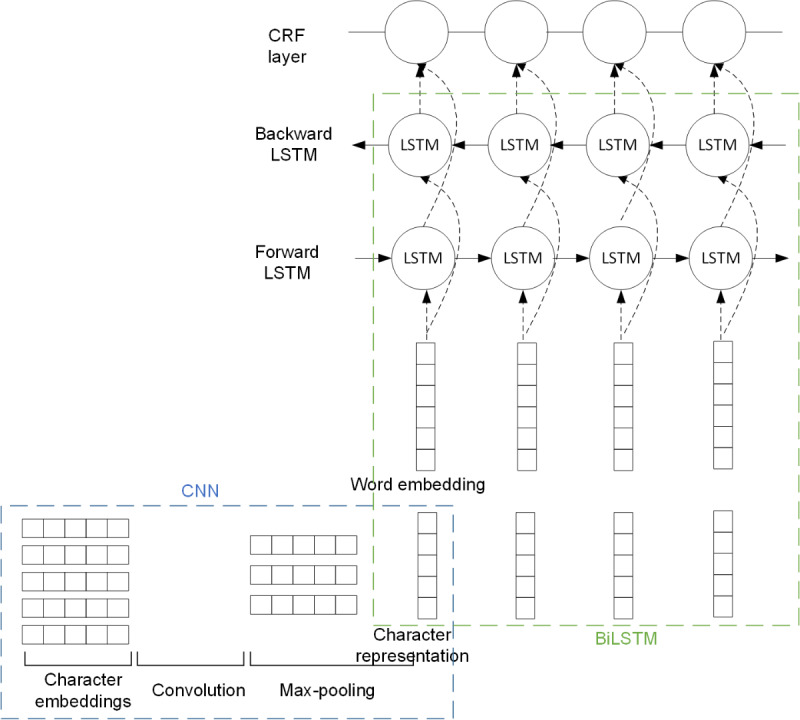
Bidirectional long short-term memory (BiLSTM), convolutional neural networks (CNNs), and conditional random field (CRF) neural network architecture.

MediReader uses the so-called *BiLSTM-CNN-CRF* deep learning neural tagging network based on works of Lample et al [27] and Ma et al [28]. This network combines bidirectional long short-term memory (BiLSTM) [29], convolutional neural networks (CNNs) [30], and conditional random field (CRF) [31] to enable effective entity recognition. The overall architecture of the proposed neural network is shown in Figure 2.

Word embedding [32] is used to transform words into low-dimensional vectors, so that the semantics of words and relationships between them can be captured. In our model, we use publicly available pretrained word embeddings from large medical corpora to accurately represent the meaning of each entity in the medical and health care domain. The word embeddings we used included global vectors embeddings [33] and a new embedding we generated using concepts that we extracted from the MedMentions data set [34]. In addition to word embedding, character-level embedding was used to represent input tokens. A CNN was used to encode the character-level information of a word.

For each word, the character-level representation was computed by the CNN with character embeddings as inputs. Then, the combined character-level and word-level encoding were fed into a BiLSTM to model the context information of each word. LSTMs [29] are variants of recurrent neural network [35], designed to cope with the gradient vanishing problems of recurrent neural network. A total of 2 LSTMs were used so that each sequence can be presented forward and backward to 2 separate hidden states to capture both the past and future information, respectively. Then, the 2 hidden states were concatenated to generate the final output. Finally, the output vectors of BiLSTM were fed into a CRF layer to jointly decode the best labels for the whole sentence.

### Medical Entity Linking

After the medical entities (referred to as *mentions* in this section) were identified from a document, they were mapped into appropriate entities defined in the knowledge base that has rich information describing the mentions and their relationships with other entities. Then, the entities defined in the knowledge base can be used to explain the mentions in the document. Owing to of text ambiguity, the same mention can often refer to many different entities depending on the context, as many entity names tend to be polysemous. This task was executed in two steps, namely, candidate generation and candidate ranking.

To link mentions to the right entities defined in a knowledge base, the system needs to generate a manageable candidate list containing possible entities that the mention may refer to. In our system, the knowledge base entries were retrieved from a subset of the UMLS concepts data set and extended using Wikidata [9]. Wikidata is a multidisciplinary ontological database that encompasses many medicine-related entries, such as human genes, human proteins, diseases, drugs, drug classes, therapies, and so on. All these items are connected to create an extensive biomedical taxonomy using taxonomic Wikidata properties [36]. Wikidata was used as a secondary database, relying mainly on other resources to match its content. Wikidata connects with UMLS through its concept unique identifier. We used the taxonomic properties of Wikidata, such as the instance of (P31), subclass of (P279), part of (P361), and has part (P527), to extend an entity.

Entities (concepts and aliases) in the knowledge base were encoded using term frequency-inverse document frequency scores of character n-grams (n=3 in our implementation) that appears more than a certain number of times in the knowledge base. Then, the k-nearest neighbor search was applied to generate candidate entities for linking a given mention.

Entity linking may encounter the problem of entity ambiguity; that is, 1 mention may be mapped to several candidate entries in the knowledge base. For example, the word *cold* has multiple meanings even in the medical domain including *common cold*, *cold sensation*, and *chronic obstructive airway disease (cold)* [37]. In the candidate ranking phase, we disambiguated the candidate entities using the word sense disambiguation system proposed by Stevenson et al [38]. This system leverages the context in the text and combines various types of information including linguistic features and knowledge sources specific to the biomedical domain. The domain-independent linguistic features include local collocations and salient bigrams and unigrams. For knowledge sources, UMLS concept unique identifier and Medical Subject Headings were considered. Vector space model [39] was used as the learning model.

### Personalized Annotation

After mentions in the document and entities in the knowledge sources were linked, annotation was performed. Annotating all medicine-related mentions is unnecessary as readers may know many of them. By contrast, a full annotation may cause discomfort to readers. Therefore, the system needs to determine which mentions should be annotated. MediReader proposes a personalized annotation scheme that annotates a mention based on an individual reader’s health literacy level, as discussed in the previous section. For readers with very low literacy levels, more mentions should be annotated, and the annotation should be easy to understand. For readers with high literacy levels, only complex medical terms should be annotated.

Medical term’s difficulty and readability assessment was approached as a classification problem. We used a feature set with many features commonly used for standard natural language processing, such as grammatical metrics, semantic metrics, and new composite metrics. We also added new features to the biomedical domain to make the classification specialized in this field. The feature set included the following items:

1. Syntactic categories; for example, nouns, adjectives, proper names, verbs, and abbreviations
2. Number of characters and syllables in the word
3. Prefixes and suffixes of the word
4. Number and percentage of consonants, vowels, and other characters (ie, hyphen, apostrophe, and commas)
5. Presence of words in WordNet
6. Word frequency in Google
7. Word frequency in UMLS
8. Word semantic categories in UMLS
9. Pretrained word embeddings using MedMentions

To build our data set, we extracted medical concepts from the website of Medical Transcription Samples [40], which contains a vast collection of transcribed medical transcription sample reports of many specialties. We used the data set to train a prediction model that again used the BiLSTM-CNN model. We extracted 1000 terms from the website. We used 6 graduate students (n=1, 17% native English speaker and n=5, 83% nonnative speakers) to identify whether they can understand the meaning of each of the 1000 words. If a word received 6 positive answers, it was labeled as easy. If it received 5 or 4 positive answers, it was labeled as medium. If it received <4 positive answers, it was labeled as difficult. These labeled terms were used to train the classification system.

On the basis of a reader’s health literacy level, medical mentions were annotated. For readers with high health literacy levels, only difficult words were annotated. For readers with low health literacy levels, medium and difficult words were annotated. We did not annotate easy words such as *fever*, *wound*, *operation*, and so on. In addition, medical stop words were removed before the entity linking process.

**Figure 3:**
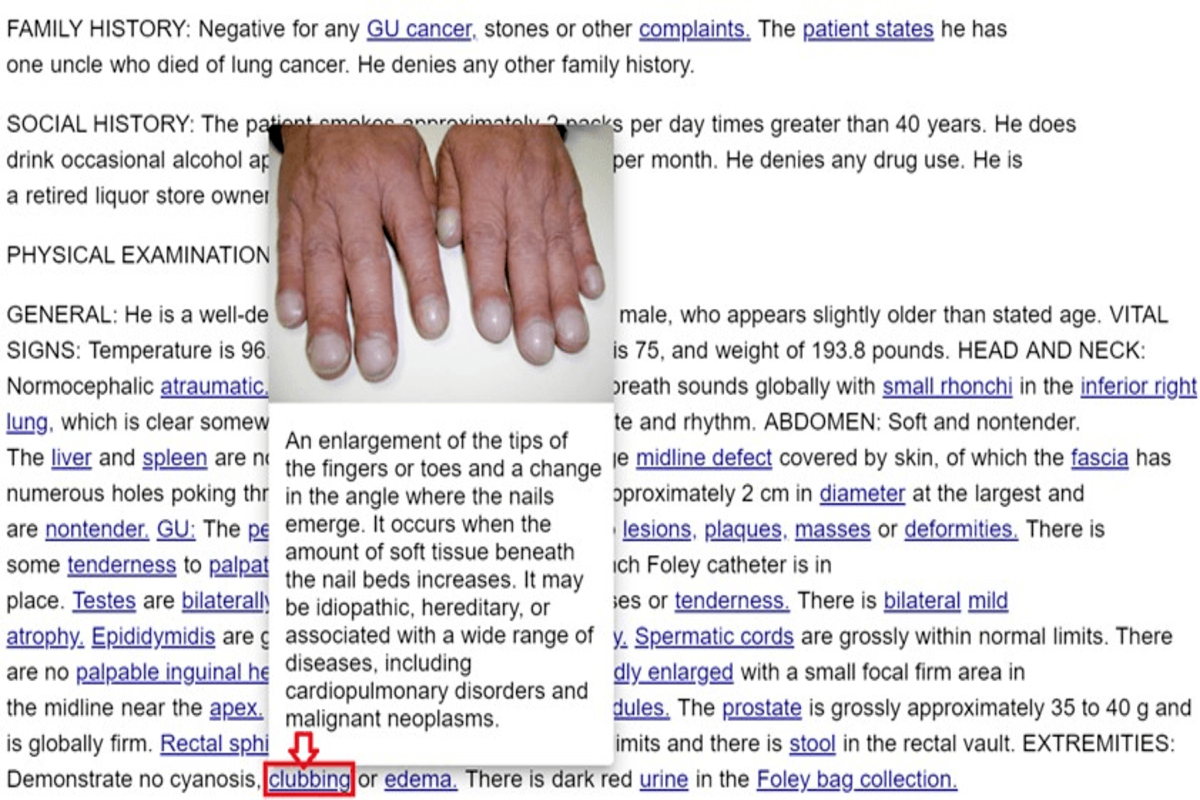
Screenshot of an annotated document.

Each entity was annotated with its definition in the knowledge source. In addition, they were linked by taxonomic relations, such as *instance of*, *subclass of*, and *part of* and major nontaxonomic associative relations (eg, *drug used for treatment* and *risk factor*) to allow a reader to better understand the various aspects about the concept. Figure 3 shows a screenshot of an annotated document for readers with low health literacy levels.

## Results

### Test Setup

We implemented the MediReader prototype system as a mobile app. We conducted a set of evaluation tests with representative users to assess the technical viability and effectiveness of this app. To conduct the test, we developed a test plan, recruited participants, and then, analyzed and reported our findings. In our study, we used 2 types of quality metrics that combine to form the big construct we call *usability*. One type of metric was objective criteria, and the other type was subjective criteria.

For the *objective* quality measurement, we invited participants to use MediReader and created a set of tasks for them to complete. Then, we recorded the time they spent on the tasks, their success rates, and errors. For comparison, a control group was also used to perform the same tasks but without the help of MediReader. In our test, we used the same participants to act as both the experimental and control groups. Specifically, the task was to ask users to read 2 sets of medical documents; each set contains 3 physicians’ notes on three different diseases, namely, endometrial adenocarcinoma, bladder cancer, and breast calcifications. We tried to choose common and familiar diseases that involve unfamiliar vocabularies. Cancer is a familiar disease. However, many cancer-related documents are difficult to understand. Therefore, we chose two types of cancer: endometrial adenocarcinoma (uterine cancer) and bladder cancer. Breast calcifications are common among women; thus, we chose it as the third disease. One set of documents was annotated using MediReader, and the other set was original medical documents without any annotation. Each set of documents contained 12 questions related to the notes to identify whether the participants can understand the notes. All questions were multiple-choice with 3 answers, and only 1 of them was correct. These notes focused on different diseases and treatments and were randomly selected from real-world web-based physician notes [40]. For a particular participant, one set of documents was randomly selected and annotated using our app, and the other set of documents was shown to the participants without any annotation. In this way, we created a control group that read the same physician notes as the experimental group and answered the same set of questions, but without the help of MediReader. Before the task, health literacy tests were conducted to assess the participants’ health literacy skills (high and low only).

We also performed a *subjective* evaluation of the system through a user satisfaction survey. We surveyed participants with 6 satisfactory questions after they used our MediReader prototype system. All the questions were measured using a 4-point Likert scale that ranged from strongly disagree (rating=1) to strongly agree (rating=4).

Before conducting the test, we conducted a pilot study to verify our programming, database, and scoring. We expected that some participants may not read the assigned documents and questions and may choose random answers. To eliminate such responses, we included qualifying questions in different sections. In each multiple-choice section, we added 1 question that could easily be answered correctly if the participant read it. Participants who did not answer these questions correctly were eliminated from the data set.

### Test Outcome

Owing to the difficulty in recruiting participants, we had to include as many participants as possible. Therefore, we only required the participants who were aged ≥18 years and knew English. A total of 52 individuals participated in our test. Among the 52 individuals, 13 (25%) individuals did not complete the test and 11 (21%) individuals were disqualified based on our qualifying questions. The remaining 54% (28/52) of the participants completed the test successfully. Table 1 shows the basic demographic information about the participants.

**Table 1:** Demographic information of the participants (N=28).

Variable			Value, n (%)
**Sex**			
	Men	13 (46)	
	Women	15 (54)	
**Age (years)**			
	40-50	4 (14)	
	30-39	7 (25)	
	20-29	17 (61)	
**Education**			
	Undergraduate	19 (68)	
	Postgraduate	9 (32)	
**Health literacy level**			
	Low	10 (36)	
	High	18 (64)	

We compared the average scores between the experimental and control groups for all the questions. We noticed that the experimental group significantly exceeded the control group, as they scored 76% compared with 36% for the control group, which means that participants who were provided with medical documents annotated using our tool had a higher score than those who were given documents without annotation.

In terms of the time spent for the reading test, we found that the experimental group spent more time than the control group (29 minutes and 24 minutes, respectively). From the participants’ comments, we learned that they spent time in reading more information about the annotated terms and other information related to the term. We believe that this explains why the experimental group spent more time in the test.

Table 2 demonstrates that the contents of the medical documents affect the participants’ reading and impact our tool’s performance. For example, regarding the first type of document, that is, the document about endometrial adenocarcinoma (disease 1 in Table 2), the control group obtained a score of approximately 60% when they read unannotated documents. However, the score moderately increased to approximately 70% for experimental groups when they read the same document annotated using our tool. For the third type of document, that is, the document about breast calcifications (disease 3 in Table 2), there was great increase (from 27% to 87%) in the scores for the experimental group compared with the control group.

**Table 2:** Comparison of the average scores of the experimental and control groups on different medical domains or diseases.

Disease and group			Score, mean (SD)
**Disease 1**			
	Experimental	70 (0.35)	
	Control	60 (0.32)	
**Disease 2**			
	Experimental	74 (0.39)	
	Control	26 (0.29)	
**Disease 3**			
	Experimental	87 (0.19)	
	Control	27 (0.16)	

Our tool has a different impact on participants with different health literacy levels. The average score increased from 36% to 88% for participants with high health literacy levels. For participants with low health literacy, the score greatly increased from 17% to 85%.

Table 3 shows the detailed scoring of the experimental and control groups with different health literacy levels for different medical subjects. The scores increased for the participants in the experimental group, who read annotated medical reports regardless of their level of health literacy. For documents on endometrial adenocarcinoma (disease 1), the score for the participants with low literacy in the experimental group showed a moderate increase of approximately 20%; the increase was lower (10%) for participants with high health literacy. The experimental group showed great increase in the average score for the questions related to bladder cancer (disease 2) and breast calcifications (disease 3) for participants with both high and low health literacy levels. The score for participants with low health literacy increased considerably from 12.5% in the control group to approximately 61% in the experimental group for documents related to bladder cancer (disease 2). The score for participants with high health literacy increased from approximately 43% in the control group to approximately 86% in the experimental group for the same type of documents. Similarly, for questions about breast calcifications (disease 3), the average score increased from 36% to 88% for participants with high literacy and from 17% to 85% for participants with low literacy.

We applied the Wilcoxon rank-sum test [41] to determine the differences between the experimental and control groups. *P* value <.05 was considered as significant. Regarding endometrial adenocarcinoma (disease 1) document, the difference between the experimental group and control group was not significant (*P*=.54 for participants with high literacy and *P*=.20 for participants with low literacy). On the other hand, there was significant difference in the score for the participants who solved questions on disease 3 (breast calcifications; *P*=.002 for participants with high literacy and *P*=.002 for participants with low literacy). For participants who dealt with the document about bladder cancer (disease 2), there was significant annotation effect only on users with high health literacy (*P*=.02); for participants with low health literacy, the difference was not significant (*P*=.06).

Table 3 summarizes the detailed scoring of the experimental and control groups and shows the *P* values.

To identify participants’ overall satisfaction, they were asked to provide their satisfaction feedback regarding the use of the mobile app. The participant satisfaction analysis showed that, in general, the participants were satisfied with the mobile app. As shown in Table 4, most participants agreed (18/28, 64% strongly agreed, and 6/28, 21% agreed) that the app helped them understand the medical documents better. Only 14% (4/28) of the participants disagreed (1/28, 4% strongly disagreed, and 3/28, 10% disagreed). Similarly, as shown in Table 4, most participants agreed that the app was easy to use and that they would recommend it. Regarding whether appropriate medical terms were annotated, 43% (12/28) of the participants strongly agreed, and 46% (13/28) of the participants agreed that the app annotated medical terms, as shown in Table 4.

**Table 3:** Comparison of the average score and *P* values of the experimental and control groups with different health literacy levels on different medical domains or diseases.

Disease, health literacy level, and group				Score, mean (SD)		*P* value
**Disease 1**						
	**High**					.54
		Experimental	71 (0.30)			
		Control	62 (0.23)			
	**Low**					.20
		Experimental	67 (0.42)			
		Control	46 (0.25)			
**Disease 2**						
	**High**					.02
		Experimental	86 (0.26)			
		Control	43 (0.25)			
	**Low**					.06
		Experimental	61 (0.49)			
		Control	12.5 (0.25)			
**Disease 3**						
	**High**					.002
		Experimental	88 (0.16)			
		Control	36 (0.15)			
	**Low**					.002
		Experimental	85 (0.23)			
		Control	17 (0.11)			

**Table 4:** Overall feedback regarding the use of the mobile app (N=28).

Survey question	Strongly agreed, n (%)	Agreed, n (%)	Disagreed, n (%)	Strongly disagreed, n (%)
The application helped me understand medical documents better	18 (64)	6 (21)	3 (10)	1 (4)
The application was easy to use	18 (64)	4 (14)	4 (14)	1 (4)
I will recommend the application to others	18 (64)	6 (21)	3 (11)	1 (4)
The application annotated appropriate medical terms	12 (43)	13 (46)	2 (7)	1 (4)

## Discussion

### Principal Findings

People need to understand medical information to have the best chance of a good health outcome. However, understanding medical information is more difficult than what most people realize, as it requires a certain degree of health literacy. To assist people in understanding medical documents, we designed, developed, and evaluated a mobile app, MediReader. MediReader uses external knowledge sources to annotate medical documents according to each user’s health literacy level. Algorithms based on machine learning and natural language processing have been proposed and implemented to recognize medical entities, identify the complexity of medical terms, and link medical terms to external knowledge that can explain the terms. MediReader was evaluated through task-based user studies with a control group and users’ satisfaction survey.

On the basis of the comparison with a control group, the test results demonstrate that MediReader can improve users’ understanding of medical documents. This improvement is particularly significant for users with low health literacy levels. The satisfaction survey shows that users are satisfied with the tool in general. The result also shows that some medical information is more difficult to understand than others, even with the help of MediReader. In summary, our study demonstrated that it is feasible and effective to implement an mHealth tool to help people better understand medical documents.

MediReader simplified medical documents for the general public and improved their understanding, whereas most existing annotation tools, such as MetaMap [42] and Clinical Text Analysis and Knowledge Extraction System [43], were designed for medical professionals such as physicians, medical students, and biomedical researchers. It is not clear how these tools will benefit the general users. MediReader adapts its interface based on users’ health literacy, whereas most existing tools (eg, National Center for Biomedical Ontology Annotator [44] and BioMedical Concept Annotation System [45]) do not distinguish between users. MediReader uses an effective machine learning mechanism to locate medical terms and subsequently link and explain medical terms that are most appropriate for the given context. Many existing systems (such as National Center for Biomedical Ontology Annotator [44] and ConceptMapper [46]) have adopted the dictionary-based matching that lacks disambiguation ability; they only list all meanings of the annotated entity.

### Limitations and Future Work

This study had some limitations. The qualitative evaluation was performed with limited participants and most of them were college students. The results that will be obtained if it is conducted on underrepresented racial or ethnic groups and older adults remains questionable. More comprehensive user studies will be performed on a large population to evaluate the usability, satisfaction rate of users, and health and quality of life-improvement outcomes.

Some medical information is still difficult to understand even after our tool’s annotation.

Through our test, we found that some medical terms are annotated with annotations and definitions that are difficult to understand, especially when the annotations are retrieved from professional medical resources such as the UMLS vocabularies. We will work on exploiting more information sources (eg, Google Knowledge Graph) to enrich and simplify the annotation.

When a new document is loaded, there is a delay in providing the annotations to the users. We will continue to optimize our algorithms in natural language processing and machine learning to reduce the execution time. In addition, we plan to encode frequently used knowledge and store it in the storage memory of the device to further reduce the delay.

### Conclusions

Limited health literacy may restrict an individual’s participation in health contexts and activities. To help people improve their health literacy and understand medical documents better, in this study, we proposed and evaluated an mHealth app, MediReader. The app annotates medical documents with information that people can understand. Our experiments demonstrated that this tool can help users better comprehend the contents of medical documents. It is especially useful for people with low health literacy levels. From our test, we found that low health literacy does not necessarily correspond to general low literacy; individuals who may be extremely literate in their areas of expertise (eg, graduate students) may also have a problem in understanding medical terminology. Further research is needed to overcome the limitations of this study.

## Abbreviations

BiLSTM: bidirectional long short-term memory
CNN: convolutional neural network
CRF: conditional random field
EHR: electronic health record
IRT: item response theory
mHealth: mobile health
UMLS: Unified Medical Language System
